# 3-Amino­pyridinium picrate

**DOI:** 10.1107/S1600536810037190

**Published:** 2010-09-30

**Authors:** Yan-jun Li

**Affiliations:** a College of Chemical Engineering and Technology, Wuhan University of Science and Technology, Wuhan 430081, People’s Republic of China

## Abstract

During the formation of the title compound, C_5_H_7_N_2_^+^·C_6_H_2_N_3_O_7_^−^, a phenolic proton is transferred to the pyridine N atom. In the crystal structure, the ions are linked by inter­molecular N—H⋯O and N—H⋯(O,O) hydrogen bonds into layers running parallel to (100). These layers are connected by weak π–π stacking inter­actions between symmetry-related pyridine and picric benzene rings with a centroid–centroid distance of 3.758 (2) Å, forming a three-dimensional network.

## Related literature

For applications of picric acid derivatives, see: Pascard *et al.* (1982 ▶); Pearson *et al.* (2007 ▶); Shakir *et al.* (2009 ▶). For a related structure, see: Harrison *et al.* (2007 ▶).
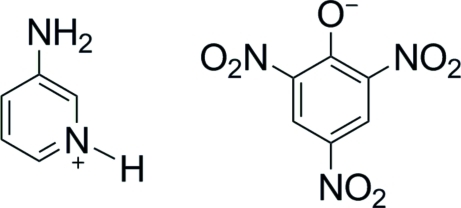


## Experimental

### 

#### Crystal data

C_5_H_7_N_2_^+^·C_6_H_2_N_3_O_7_^−^
*M* *_r_* = 323.23
Monoclinic, 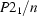

*a* = 8.2174 (8) Å
*b* = 13.5842 (13) Å
*c* = 11.8218 (12) Å
β = 102.117 (2)°
*V* = 1290.2 (2) Å^3^
*Z* = 4
Mo *K*α radiation
μ = 0.14 mm^−1^
*T* = 297 K
0.45 × 0.05 × 0.02 mm

#### Data collection

Bruker SMART CCD diffractometer
Absorption correction: multi-scan (*SADABS*; Sheldrick, 1996 ▶) *T*_min_ = 0.939, *T*_max_ = 0.997
14192 measured reflections
2804 independent reflections
1391 reflections with *I* > 2σ(*I*)
*R*_int_ = 0.072

#### Refinement

*R*[*F* ^2^ > 2σ(*F* ^2^)] = 0.060
*wR*(*F* ^2^) = 0.162
*S* = 1.03
2804 reflections
217 parameters
H atoms treated by a mixture of independent and constrained refinement
Δρ_max_ = 0.34 e Å^−3^
Δρ_min_ = −0.24 e Å^−3^

### 

Data collection: *SMART* (Bruker, 2001 ▶); cell refinement: *SAINT* (Bruker, 2001 ▶); data reduction: *SAINT*; program(s) used to solve structure: *SHELXS97* (Sheldrick, 2008 ▶); program(s) used to refine structure: *SHELXL97* (Sheldrick, 2008 ▶); molecular graphics: *PLATON* (Spek, 2009 ▶); software used to prepare material for publication: *SHELXTL* (Sheldrick, 2008 ▶).

## Supplementary Material

Crystal structure: contains datablocks I, global. DOI: 10.1107/S1600536810037190/lh5128sup1.cif

Structure factors: contains datablocks I. DOI: 10.1107/S1600536810037190/lh5128Isup2.hkl

Additional supplementary materials:  crystallographic information; 3D view; checkCIF report

## Figures and Tables

**Table 1:** Hydrogen-bond geometry (Å, °)

*D*—H⋯*A*	*D*—H	H⋯*A*	*D*⋯*A*	*D*—H⋯*A*
N5—H5⋯O7	0.91 (3)	1.79 (3)	2.607 (3)	148 (3)
N5—H5⋯O6	0.91 (3)	2.46 (3)	3.179 (4)	136 (3)
N4—H4*B*⋯O6^i^	0.86 (4)	2.48 (4)	3.119 (4)	132 (3)
N4—H4*A*⋯O3^ii^	0.85 (4)	2.44 (4)	3.172 (4)	145 (3)

Symmetry codes: (i) 
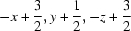
; (ii) 
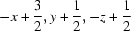
.

**Fig. 1.**
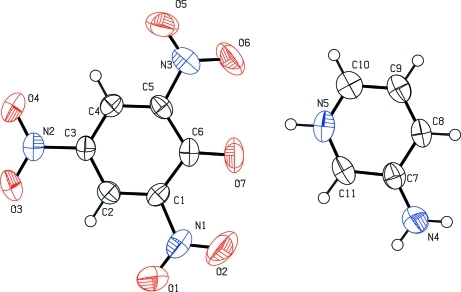
The asymmetric unit of the title compound with displacement ellipsoids drawn at the 30% probability level.

**Fig. 2.**
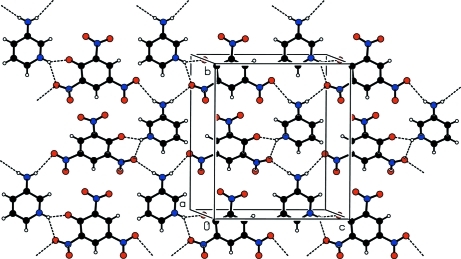
Part of the crystal structure with hydrogen bonds shown as dashed lines. For the sake of clarity, the H atoms not involved in the hydrogen-bonds pattern have been omitted.

## supplementary crystallographic information

### Comment

Picric acid has been used in the characterization of organic bases because of the ease of crystallization and hence purification when picrate derivatives are produced (Pascard *et al.*, 1982; Pearson *et al.*, 2007; Harrison *et al.*, 2007; Shakir *et al.*, 2009). Here, we report the crystal structure of the title salt.

In the title compound, a hydrogen atom has been transferred from the picric acid molecule to the nitrogen atom of the pyridine ring and hence a 1:1 organic is formed salt (Fig.1). In the picric acid molecule, the geometric parameters of C6—O7 = 1.236 (3)Å and C1—C6—C5 = 112.0 (2)° confirm the transfer of the proton.

In the crystal structure, the molecular components are linked into a two dimensional zigzag-like layers (Fig.2) running parallel to (110) by intermolecular N—H···O hydrogen bonds (Table 1). These adjacent (100) layers are linked by weak π–π interaction between symmetry related pyridine and picric benzene rings (centroid-to-centroid distance = 3.758 (2) Å, symmetry code: 2 - *x*, 1 - *y*, 1 - *z*) into a three-dimensional network.

### Experimental

Picric acid (0.69 g, 3 mmol) and 3-aminopyridine (0.28 g, 3 mmol) were mixed in 10 ml ethanol. The mixture was kept at room temperature for two weeks. Yellow needeles suitable for single-crystal X-ray diffraction were obtained at the bottom of the vessel.

### Refinement

The carbon-bound hydrogen atoms were placed in ideal positions with C—H = 0.93Å and *U*_iso_(H) = 1.2U_eq_(C). The nitrogen-bound H atoms were located in a difference map and refined with *U*_iso_(H) = 0.092Å^2^.

### Crystal data

**Table:**

C_5_H_7_N_2_^+^·C_6_H_2_N_3_O_7_^−^	*F*(000) = 664
*M**_r_* = 323.23	*D*_x_ = 1.664 Mg m^−^^3^
Monoclinic, *P*2_1_/*n*	Mo *K*α radiation, λ = 0.71073 Å
Hall symbol: -P 2yn	Cell parameters from 1100 reflections
*a* = 8.2174 (8) Å	θ = 2.3–20.4°
*b* = 13.5842 (13) Å	µ = 0.14 mm^−^^1^
*c* = 11.8218 (12) Å	*T* = 297 K
β = 102.117 (2)°	Needle, yellow
*V* = 1290.2 (2) Å^3^	0.45 × 0.05 × 0.02 mm
*Z* = 4	

### Data collection

**Table:**

Bruker SMART CCD diffractometer	2804 independent reflections
Radiation source: fine-focus sealed tube	1391 reflections with *I* > 2σ(*I*)
graphite	*R*_int_ = 0.072
φ and ω scans	θ_max_ = 27.0°, θ_min_ = 2.3°
Absorption correction: multi-scan (*SADABS*; Sheldrick, 1996)	*h* = −10→10
*T*_min_ = 0.939, *T*_max_ = 0.997	*k* = −17→17
14192 measured reflections	*l* = −15→15

### Refinement

**Table:**

Refinement on *F*^2^	Primary atom site location: structure-invariant direct methods
Least-squares matrix: full	Secondary atom site location: difference Fourier map
*R*[*F*^2^ > 2σ(*F*^2^)] = 0.060	Hydrogen site location: inferred from neighbouring sites
*wR*(*F*^2^) = 0.162	H atoms treated by a mixture of independent and constrained refinement
*S* = 1.03	*w* = 1/[σ^2^(*F*_o_^2^) + (0.0663*P*)^2^ + 0.0975*P*] where *P* = (*F*_o_^2^ + 2*F*_c_^2^)/3
2804 reflections	(Δ/σ)_max_ < 0.001
217 parameters	Δρ_max_ = 0.34 e Å^−^^3^
0 restraints	Δρ_min_ = −0.24 e Å^−^^3^

### Special details

**Table:**

Geometry. All e.s.d.'s (except the e.s.d. in the dihedral angle between two l.s. planes) are estimated using the full covariance matrix. The cell e.s.d.'s are taken into account individually in the estimation of e.s.d.'s in distances, angles and torsion angles; correlations between e.s.d.'s in cell parameters are only used when they are defined by crystal symmetry. An approximate (isotropic) treatment of cell e.s.d.'s is used for estimating e.s.d.'s involving l.s. planes.
Refinement. Refinement of *F*^2^ against ALL reflections. The weighted *R*-factor *wR* and goodness of fit *S* are based on *F*^2^, conventional *R*-factors *R* are based on *F*, with *F* set to zero for negative *F*^2^. The threshold expression of *F*^2^ > σ(*F*^2^) is used only for calculating *R*-factors(gt) *etc*. and is not relevant to the choice of reflections for refinement. *R*-factors based on *F*^2^ are statistically about twice as large as those based on *F*, and *R*- factors based on ALL data will be even larger.

### Fractional atomic coordinates and isotropic or equivalent isotropic displacement parameters (Å^2^)

**Table:**

	*x*	*y*	*z*	*U*_iso_*/*U*_eq_	
C1	0.8262 (3)	0.49045 (18)	0.2590 (2)	0.0390 (7)	
C2	0.8816 (3)	0.45022 (19)	0.1686 (2)	0.0401 (7)	
H2	0.8614	0.4819	0.0973	0.048*	
C3	0.9673 (3)	0.36279 (18)	0.1827 (2)	0.0378 (7)	
C4	1.0039 (3)	0.31637 (19)	0.2881 (2)	0.0388 (7)	
H4	1.0636	0.2577	0.2967	0.047*	
C5	0.9518 (3)	0.35703 (19)	0.3805 (2)	0.0388 (7)	
C6	0.8524 (4)	0.4466 (2)	0.3728 (2)	0.0428 (7)	
C7	0.5593 (4)	0.6203 (2)	0.7181 (2)	0.0449 (7)	
C8	0.5870 (4)	0.5718 (2)	0.8241 (2)	0.0474 (8)	
H8	0.5482	0.5994	0.8855	0.057*	
C9	0.6702 (4)	0.4844 (2)	0.8392 (3)	0.0532 (8)	
H9	0.6866	0.4527	0.9104	0.064*	
C10	0.7300 (4)	0.4426 (2)	0.7507 (3)	0.0531 (8)	
H10	0.7872	0.3831	0.7605	0.064*	
C11	0.6221 (4)	0.5750 (2)	0.6304 (2)	0.0470 (8)	
H11	0.6070	0.6043	0.5578	0.056*	
N1	0.7366 (3)	0.58418 (19)	0.2352 (3)	0.0562 (7)	
N2	1.0162 (3)	0.31865 (19)	0.0837 (2)	0.0498 (7)	
N3	0.9978 (4)	0.3051 (2)	0.4890 (2)	0.0569 (7)	
N4	0.4760 (4)	0.7061 (2)	0.7014 (3)	0.0684 (9)	
N5	0.7032 (3)	0.4902 (2)	0.6502 (2)	0.0511 (7)	
O1	0.6984 (4)	0.61237 (17)	0.1355 (3)	0.0962 (10)	
O2	0.6942 (4)	0.6267 (2)	0.3121 (2)	0.1179 (12)	
O3	0.9816 (3)	0.36138 (17)	−0.00931 (18)	0.0755 (8)	
O4	1.0901 (3)	0.24022 (17)	0.09581 (19)	0.0736 (7)	
O5	1.1054 (3)	0.24169 (18)	0.49917 (19)	0.0764 (8)	
O6	0.9316 (3)	0.3268 (2)	0.5696 (2)	0.0916 (9)	
O7	0.7949 (3)	0.48236 (16)	0.45224 (19)	0.0770 (8)	
H4A	0.455 (5)	0.730 (3)	0.634 (3)	0.092*	
H4B	0.436 (5)	0.731 (3)	0.757 (3)	0.092*	
H5	0.749 (4)	0.465 (2)	0.592 (3)	0.092*	

### Atomic displacement parameters (Å^2^)

**Table:**

	*U*^11^	*U*^22^	*U*^33^	*U*^12^	*U*^13^	*U*^23^
C1	0.0361 (16)	0.0317 (15)	0.0490 (17)	0.0008 (12)	0.0087 (14)	−0.0052 (13)
C2	0.0394 (17)	0.0427 (16)	0.0376 (16)	−0.0036 (14)	0.0069 (13)	0.0015 (13)
C3	0.0414 (17)	0.0381 (16)	0.0369 (16)	−0.0046 (13)	0.0147 (13)	−0.0068 (12)
C4	0.0371 (16)	0.0341 (15)	0.0453 (16)	−0.0027 (13)	0.0085 (13)	−0.0026 (13)
C5	0.0419 (17)	0.0425 (16)	0.0319 (15)	−0.0109 (13)	0.0075 (13)	0.0019 (12)
C6	0.0417 (18)	0.0483 (17)	0.0422 (17)	−0.0090 (14)	0.0171 (14)	−0.0127 (14)
C7	0.0475 (18)	0.0477 (18)	0.0402 (17)	−0.0098 (15)	0.0106 (14)	−0.0062 (14)
C8	0.0508 (19)	0.0570 (19)	0.0380 (17)	−0.0061 (16)	0.0178 (14)	−0.0072 (14)
C9	0.056 (2)	0.065 (2)	0.0385 (17)	−0.0058 (17)	0.0098 (16)	−0.0019 (15)
C10	0.049 (2)	0.057 (2)	0.0526 (19)	−0.0070 (15)	0.0094 (16)	−0.0019 (16)
C11	0.0470 (19)	0.061 (2)	0.0333 (16)	−0.0152 (16)	0.0095 (14)	−0.0029 (14)
N1	0.0469 (16)	0.0525 (17)	0.0693 (19)	0.0071 (13)	0.0129 (15)	−0.0143 (15)
N2	0.0528 (17)	0.0548 (17)	0.0453 (16)	−0.0010 (13)	0.0183 (13)	−0.0087 (13)
N3	0.0603 (19)	0.0655 (18)	0.0432 (16)	−0.0104 (16)	0.0070 (14)	0.0083 (14)
N4	0.095 (2)	0.0606 (19)	0.0508 (18)	0.0098 (17)	0.0180 (17)	0.0020 (15)
N5	0.0461 (17)	0.0593 (17)	0.0514 (18)	−0.0099 (13)	0.0184 (13)	−0.0191 (14)
O1	0.127 (2)	0.0793 (18)	0.094 (2)	0.0456 (17)	0.0484 (19)	0.0347 (15)
O2	0.144 (3)	0.109 (2)	0.087 (2)	0.074 (2)	−0.0071 (19)	−0.0394 (17)
O3	0.100 (2)	0.0951 (18)	0.0367 (12)	0.0179 (15)	0.0255 (12)	0.0006 (12)
O4	0.0958 (19)	0.0592 (14)	0.0733 (16)	0.0264 (14)	0.0347 (14)	−0.0095 (12)
O5	0.098 (2)	0.0659 (16)	0.0572 (15)	0.0144 (15)	−0.0020 (14)	0.0129 (12)
O6	0.094 (2)	0.136 (2)	0.0549 (15)	0.0175 (17)	0.0390 (15)	0.0328 (15)
O7	0.106 (2)	0.0784 (16)	0.0612 (15)	0.0045 (14)	0.0509 (15)	−0.0147 (12)

### Geometric parameters (Å, °)

**Table:**

C1—C2	1.361 (3)	C8—H8	0.9300
C1—C6	1.446 (4)	C9—C10	1.369 (4)
C1—N1	1.468 (3)	C9—H9	0.9300
C2—C3	1.373 (3)	C10—N5	1.329 (4)
C2—H2	0.9300	C10—H10	0.9300
C3—C4	1.372 (4)	C11—N5	1.328 (4)
C3—N2	1.445 (3)	C11—H11	0.9300
C4—C5	1.371 (3)	N1—O2	1.190 (3)
C4—H4	0.9300	N1—O1	1.216 (3)
C5—N3	1.443 (3)	N2—O4	1.220 (3)
C5—C6	1.457 (4)	N2—O3	1.223 (3)
C6—O7	1.236 (3)	N3—O5	1.222 (3)
C7—N4	1.345 (4)	N3—O6	1.229 (3)
C7—C8	1.392 (4)	N4—H4A	0.85 (4)
C7—C11	1.394 (4)	N4—H4B	0.86 (4)
C8—C9	1.364 (4)	N5—H5	0.91 (3)
			
C2—C1—C6	123.8 (2)	C8—C9—C10	120.8 (3)
C2—C1—N1	115.8 (3)	C8—C9—H9	119.6
C6—C1—N1	120.4 (3)	C10—C9—H9	119.6
C1—C2—C3	120.0 (3)	N5—C10—C9	117.5 (3)
C1—C2—H2	120.0	N5—C10—H10	121.2
C3—C2—H2	120.0	C9—C10—H10	121.2
C4—C3—C2	121.1 (2)	N5—C11—C7	120.2 (3)
C4—C3—N2	120.0 (2)	N5—C11—H11	119.9
C2—C3—N2	118.9 (2)	C7—C11—H11	119.9
C5—C4—C3	119.5 (3)	O2—N1—O1	122.0 (3)
C5—C4—H4	120.2	O2—N1—C1	119.3 (3)
C3—C4—H4	120.2	O1—N1—C1	118.4 (3)
C4—C5—N3	116.4 (3)	O4—N2—O3	122.4 (2)
C4—C5—C6	123.4 (2)	O4—N2—C3	118.9 (3)
N3—C5—C6	120.2 (3)	O3—N2—C3	118.6 (3)
O7—C6—C1	122.6 (3)	O5—N3—O6	121.5 (3)
O7—C6—C5	125.4 (3)	O5—N3—C5	118.8 (3)
C1—C6—C5	112.0 (2)	O6—N3—C5	119.7 (3)
N4—C7—C8	121.6 (3)	C7—N4—H4A	118 (3)
N4—C7—C11	122.0 (3)	C7—N4—H4B	119 (3)
C8—C7—C11	116.4 (3)	H4A—N4—H4B	122 (4)
C9—C8—C7	120.8 (3)	C11—N5—C10	124.2 (3)
C9—C8—H8	119.6	C11—N5—H5	117 (2)
C7—C8—H8	119.6	C10—N5—H5	118 (2)
			
C6—C1—C2—C3	0.3 (4)	C7—C8—C9—C10	0.6 (4)
N1—C1—C2—C3	179.8 (2)	C8—C9—C10—N5	−0.2 (4)
C1—C2—C3—C4	−2.4 (4)	N4—C7—C11—N5	−179.8 (3)
C1—C2—C3—N2	176.4 (2)	C8—C7—C11—N5	0.0 (4)
C2—C3—C4—C5	1.1 (4)	C2—C1—N1—O2	−175.1 (3)
N2—C3—C4—C5	−177.7 (2)	C6—C1—N1—O2	4.4 (4)
C3—C4—C5—N3	−178.8 (2)	C2—C1—N1—O1	9.8 (4)
C3—C4—C5—C6	2.4 (4)	C6—C1—N1—O1	−170.7 (3)
C2—C1—C6—O7	−176.9 (3)	C4—C3—N2—O4	−0.1 (4)
N1—C1—C6—O7	3.6 (4)	C2—C3—N2—O4	−178.9 (3)
C2—C1—C6—C5	2.8 (4)	C4—C3—N2—O3	179.8 (3)
N1—C1—C6—C5	−176.7 (2)	C2—C3—N2—O3	1.0 (4)
C4—C5—C6—O7	175.5 (3)	C4—C5—N3—O5	14.6 (4)
N3—C5—C6—O7	−3.2 (4)	C6—C5—N3—O5	−166.5 (3)
C4—C5—C6—C1	−4.1 (4)	C4—C5—N3—O6	−167.1 (3)
N3—C5—C6—C1	177.1 (2)	C6—C5—N3—O6	11.8 (4)
N4—C7—C8—C9	179.3 (3)	C7—C11—N5—C10	0.4 (4)
C11—C7—C8—C9	−0.5 (4)	C9—C10—N5—C11	−0.3 (4)

### Hydrogen-bond geometry (Å, °)

**Table:**

*D*—H···*A*	*D*—H	H···*A*	*D*···*A*	*D*—H···*A*
N5—H5···O7	0.91 (3)	1.79 (3)	2.607 (3)	148 (3)
N5—H5···O6	0.91 (3)	2.46 (3)	3.179 (4)	136 (3)
N4—H4B···O6^i^	0.86 (4)	2.48 (4)	3.119 (4)	132 (3)
N4—H4A···O3^ii^	0.85 (4)	2.44 (4)	3.172 (4)	145 (3)

Symmetry codes: (i) −*x*+3/2, *y*+1/2, −*z*+3/2; (ii) −*x*+3/2, *y*+1/2, −*z*+1/2.

## References

[bb1] Bruker (2001). *SMART* and *SAINT-Plus* Bruker AXS Inc., Madison, Wisconsin, USA.

[bb2] Harrison, W. T. A., Ashok, M. A., Yathirajan, H. S. & Narayana Achar, B. (2007). *Acta Cryst.* E**63**, o3277.

[bb3] Pascard, C., Riche, C., Cesario, M., Kotzyba-Hibert, F. & Lehn, J. M. (1982). *Chem. Commun.* pp. 557–558.

[bb4] Pearson, W. H., Kropf, J. E., Choy, A. L., Lee, I. Y. & Kampf, J. W. (2007). *J. Org. Chem.* **72**, 4135–4148. 10.1021/jo0703799 17465572

[bb5] Shakir, M., Kushwaha, S. K., Maurya, K. K., Arora, M. & Bhagavannarayana, G. (2009). *J. Cryst. Growth*, **311**, 3871–3875.

[bb6] Sheldrick, G. M. (1996). *SADABS*, University of Göttingen, Germany.

[bb7] Sheldrick, G. M. (2008). *Acta Cryst.* A**64**, 112–122. 10.1107/S0108767307043930 18156677

[bb8] Spek, A. L. (2009). *Acta Cryst.* D**65**, 148–155. 10.1107/S090744490804362X PMC2631630 19171970

